# Can a Small Intestine Segment Be an Alternative Biological Conduit for Peripheral Nerve Regeneration?

**DOI:** 10.4274/balkanmedj.2015.1601

**Published:** 2017-05-15

**Authors:** Mehmet S. Arda, Emre A. Koçman, Emre Özkara, Erdem Söztutar, Orhan Özatik, Aydan Köse, Cengiz Çetin

**Affiliations:** 1 Department of Pediatric Surgery, Eskişehir Osmangazi University School of Medicine, Eskişehir, Turkey; 2 Department of Plastic Reconstructive and Esthetic Surgery, Eskişehir Osmangazi University School of Medicine, Eskişehir, Turkey; 3 Department of Anatomy, Eskişehir Osmangazi University School of Medicine, Eskişehir, Turkey; 4 Department of Neurosurgery, Eskişehir Osmangazi University School of Medicine, Eskişehir, Turkey; 5 Department of Histology and Embryology, Ahi Evran University School of Medicine, Kırşehir, Turkey

**Keywords:** Peripheral nerve injury, small intestine, conduits

## Abstract

**Background:**

Autologous nerve grafts are used to bridge peripheral nerve defects. Limited sources and donor site morbidity are the major problems with peripheral nerve grafts. Although various types of autologous grafts such as arteries, veins and muscles have been recommended, an ideal conduit has not yet been described.

**Aims:**

To investigate the effectiveness of a small intestinal conduit for peripheral nerve defects.

**Study Design:**

Animal experimentation.

**Methods:**

Twenty-one rats were divided into three groups (n=7). Following anaesthesia, sciatic nerve exploration was performed in the Sham group. The 10 mm nerve gap was bridged with a 15 mm ileal segment in the small intestinal conduit group and the defect was replaced with orthotopic nerve in autologous nerve graft group. The functional recovery was tested monthly by walking-track analysis and the sciatic functional index. Histological evaluation was performed on the 12th week.

**Results:**

Sciatic functional index tests are better in autologous nerve graft group (-55.09±6.35); however, during follow-up, progress in sciatic functional index was demonstrated, along with axonal regeneration and innervation of target muscles in the small intestinal conduit group (-76.36±12.08) (p<0.05). In histologic sections, distinctive sciatic nerve regeneration was examined in the small intestinal conduit group. The expression of S-100 and neurofilament was observed in small intestinal conduit group but was less organised than in the autologous nerve graft group. Although the counted number (7459.79±1833.50 vs. 4226.51±1063.06 mm^2^), measured diameter [2.19 (2.15-2.88) vs. 1.74 (1.50-2.09) µm] and myelin sheath thickness [1.18 (1.09-1.44) vs. 0.66 (0.40-1.07) µm] of axons is significantly high in the middle sections of autologous nerve graft compared to the small intestinal conduit group, respectively (p<0.05), the peripheral nerve regeneration was also observed in the small intestinal conduit group.

**Conclusion:**

Small intestinal conduit should not be considered as an alternative to autologous nerve grafts in its current form; however, the results are promising. Even though the results are no better than autologous nerve grafts, with additional procedures, it might be a good alternative due to harvesting abundant sources without donor site morbidity.

Peripheral nerve injuries with segmental defects require bridging the gap between the stumps to facilitate nerve regeneration and a functional recovery. The gold standard for reconstruction is grafting with autologous nerves. However harvesting nerve graft results in donor site morbidity and the amount of source is also limited (1). To overcome the drawbacks of autologous nerve grafting, various types of biological and artificial conduits are employed to guide nerve regeneration (1,2). Recently, many artificial conduits with lower antigenicity and foreign body reactions are commercially available for clinical use. However, they are expensive and still do not afford adequate peripheral nerve regeneration (3,4,5,6). Therefore, demands on biological conduits are still ongoing. Autologous materials have the obvious advantages of biocompatibility and the creation of a favourable microenvironment with native extracellular matrix (ECM) and live cells that can promote peripheral nerve regeneration (7). Grafts such as vein (3,8), artery (3), muscle (9,10,11), epimysium (7) and epineurium (4,12) have been used with tubulisation techniques as nerve guide conduits. However, all of these biological conduits have their own limitations.

Tos et al. (8) stated that a nerve conduit should include an appropriate environment to support axon regeneration and should protect against scar invasion. An ideal conduit should also provide free orientation of growing axons through its lumen. During neural regeneration, it is mandatory to maintain luminal shape with a thick-walled scaffold (1). Although relatively thick-walled small intestinal sub-mucosa (SIS) was created to bridge nerve gaps in previous experimental studies (13,14,15,16), the feasibility of small intestine segments including all layers (mucosa, sub-mucosa and serosa) as a conduit for nerve gaps has not yet been investigated.

The purpose of the present study is to evaluate the efficiency of using a small intestinal conduit (SIC) in neural regeneration by comparing the outcomes with autologous nerve graft (ANG).

## MATERIALS AND METHODS

### Animals

In this study, 21 female Sprague-Dawley rats weighing 220-250 g were randomly divided into three groups (n=7), namely sham, ANG and SIC groups. The animals were housed in separate ergonomic cages with a constant temperature and air humidity and a 12 h day/night cycle. The animals had free access to standard chow and tap water until 12 h before surgery.

The study was approved by the local Ethical committee of the Eskişehir Osmangazi University (protocol #414/2014). The experiments were conducted based on the healthcare guidelines for the laboratory animals and Universal Declaration on Animal Welfare.

### Surgical procedure

**Figure 1:**
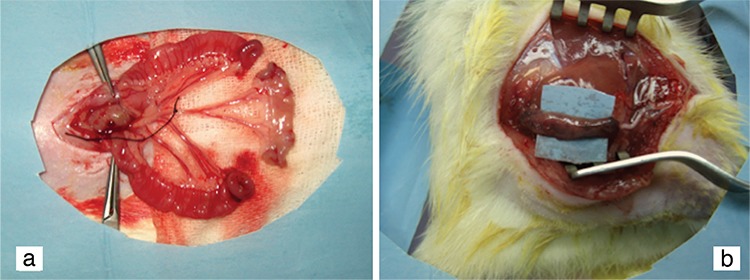
Harvesting a small intestine segment (a). Bridging the sciatic nerve gap with intestinal conduit and entabulating the proximal and distal stumps (b).

The animals received thiopental intraperitoneally at a dose of 50 mg/kg for the induction of anaesthesia. The abdominal area and left thigh were depilated and cleansed for surgery. The intestine was exposed through a small midline laparotomy incision and a 15 mm portion from the ileum was then resected (Figure 1a). The intestinal lumen was irrigated with tepid water at 24 °C. Intestinal continuity was restored with end-to-end, single layer anastomoses with 7/0 polydioxanone sutures.

In the sham-operated group, the left sciatic nerve was exposed through a gluteal muscle incision and intestinal exploration was performed with resection and anastomosis. After careful haemostasis, the inguinal and abdominal incision was sutured with resorbable 4/0 stiches. In ANG group, a 10 mm segment was excised proximal to the bifurcation of the left sciatic nerve and replaced again at the proximal and distal stumps with three stitches of 9/0 prolene sutures. In the SIC group, a 10 mm segment was also excised in a similar fashion and the gap was bridged with the resected intestinal segment. Both the proximal and distal stumps of the transected nerve were entubulated 2 mm into the intestinal conduit and fixed with three 9/0 prolene stitches (Figure 1b). Following the surgical procedure, the gluteal muscle and skin incisions were closed with 4/0 prolene in all groups. Intestinal resection and anastomosis was performed in all three groups; all individuals survived till the end of the experiment. The animals recovered for 12 weeks and were then followed up for behavioural assessments and measurements.

### Sciatic functional index assessment

A walking track study was performed at 0, 4, 8, and 12 weeks to assess the nerve regeneration progress. The animals were allowed to walk on white paper along a platform with a darkened cul-de-sac after their feet were stained with stamp ink. The normal and injured footprints were measured three times from each animal and then the sciatic functional index (SFI) was calculated using the following formula:

SFI=-38.3X (EPL-NPL)/NPL+109.5X (ETS-NTS)/NTS+13.3X (EIT-NIT)/NIT-8.8

A score of “0” indicated normal function and a score of “-100” or less represented a loss of function. Regeneration and functional gain was determined if the score was approximately restored to “0”.

### Sacrifice and sample collection

The animals were anaesthetised with ether inhalation and sacrificed via trans-cardiac blood exsanguination 12 weeks after surgery. The left thighs were re-exposed, and sciatic nerves, engrafted nerves with autogen nerves or intestinal tube complexes were extracted from the wound bed and fixed in 10% buffered formalin or 2.5% glutaraldehyde.

### Histological and morphometric analysis

The samples were embedded in paraffin after fixation in 10% buffered formalin for 24 hours. Then, 4 µm thick longitudinal sections along the neural structures or transverse sections from proximal, middle and distal areas of the specimens were obtained using a microtome (Leica RM2125, Wetzlar, Germany). After deparaffinisation and serial dehydration with ethanol, the sections were stained with toluidine blue and Masson's trichrome. Two blinded investigators using cross sections performed the quantitative histological measurements. Five random fields at X100 magnification were chosen from proximal, middle, and distal segments. The images were captured by a digital camera connected to a light-microscope (Leica DM3000, Wetzlar, Germany) and computer. Two blinded investigators made all measurements. The number of axons per 1 mm2 was estimated and axon diameter and myelin sheath thickness were measured on each sample using an image software program (Neurolucida software, MBF Bioscience, Williston, VT, USA).

### Immunohistological analysis

Unstained sections were further processed for S-100 and neurofilament immunostaining according to the manufacturer’s instructions. The slides were incubated in Tris/EDTA buffer (pH 9.0) for 5 min and then 3% hydrogen peroxide for 10 min. The slides were then washed with tris buffer saline pH 7.6 for 5 min each. Non-specific immunoreactions were blocked with ultra V-Block serum. The samples were then labelled with S-100 Protein Ab-1 (Clone 4C4.9, Thermo Scientific, Fremont, CA, USA) and neurofilament (200 kDa & 68 kDa) Ab-1 (Clone 2F11, Thermo Scientific, Fremont, CA, USA), which are antibodies for Schwann cells and axons, respectively. The samples were then stained with AEC Chromogen. The slides were counterstained with haematoxylin. The immunohistological sections were evaluated by two blinded investigators.

### Statistical analysis

IBM SPSS version 22 (IBM Corp. Released 2013. IBM SPSS Statistics for Windows, Version 22.0. Armonk, NY: IBM Corp.) was used for data analysis. The Shapiro-Wilk test of normality was used to assess compliance with the normal distribution of continuous variables in each group. Continuous normally distributed measurements (number of axons) were compared across the groups using One-Way ANOVA with the Tukey Method for multiple comparisons. The measurements that did not show a normal distribution (axon diameters and myelin sheath thickness) were compared using the Kruskal-Wallis, with Dunn Multiple Comparison test. All values are expressed as the mean ± standard deviation and median (25-75%). The significance level was set as p<0.05.

## RESULTS

### Sciatic function index results

**Figure 2:**
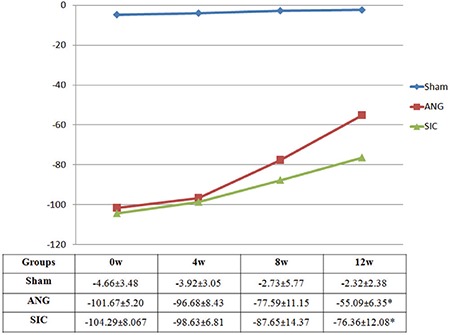
The sciatic functioning index measurement results of groups during follow up throughout 12th week, *indicates a significant difference between autologous nerve graft and small intestinal conduit (p<0.05). *SFI: sciatic functioning index; ANG: autologous nerve graft; SIC: small intestinal conduit; W: week*

The data for the sciatic nerves during the 12th week recovery period are presented in Figure 2. The SFI increased over time in the ANG and SIC groups and no functional loss was observed in the sham animals. Although SFI values at 12 weeks were significantly higher in the ANG group (-55.09±6.35) than in the SIC group (-76.36±12.08) (p<0.05), the increase in SFI values of the SIC animals indicated that some sprouting axons passed through the intestinal conduit and innervated target muscles.

### Macroscopic observations

**Figure 3:**
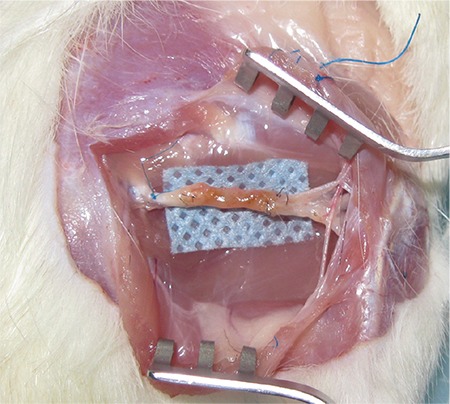
Macroscopic appearance of regenerated small intestinal conduit constructs at 12 weeks.

After the animals were sacrificed, the constructed nerves were inspected macroscopically before biopsies were performed. In the ANG group, the engrafted nerves healed uneventfully with minimal fibrosis and without neuroma formation. The constructs in the SIC group also healed well within the atrophied intestinal tubes (Figure 3). Although the intestinal covers were not trimmed or dissected to prevent injury, the midportion of the intestinal tube was dense by palpation. There were no signs of neuroma or infection (including intra-abdominal leakage, abscess, cysts etc.) observed. At the end of the experiment, when the animals sacrificed, it was observed that the donor-sites have been healed well except minor intra-abdominal adhesions.

### Histological and histomorphometric results

**Figure 4:**
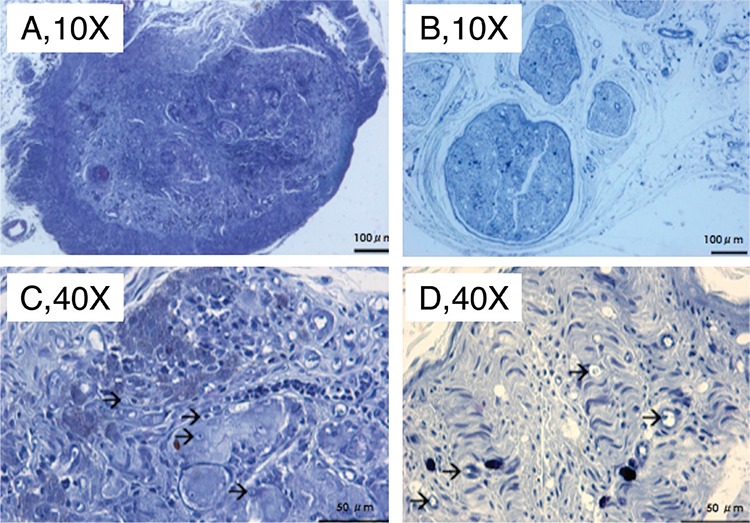
Toluidine-blue stained sections of the small intestinal conduit complex from intraconduit (a, c) and distal stump (b, d). Note the regenerated neural elements within the fibrotic tissue throughout the intestinal segment, wallerian degeneration and weak axonal regeneration in the distal stump. Magnifcations at 10X [above (a, b), Scale bar=100 μm] and 40X [below (c, d), Scale bar=50 μm]. Arrows indicate axons.

**Figure 5:**
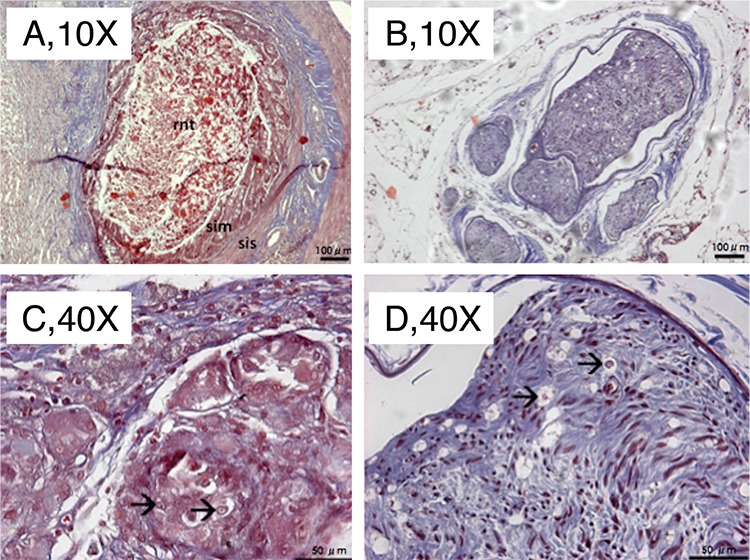
Sections from the intraconduit (a, c) and distal stump (b, d) stained with Masson's Trichrome technique. Note that rnt is stained differently from the surrounding intestinal tissue. Arrows indicate axons. rnt: regenerated neural tissue, sim: small intestinal mucosa, sis: small intestinal submucosa. Magnifcations at 10X [above (a, b), Scale bar=100 μm] and 40X [below (c, d), Scale bar=50 μm].

The sham operated group showed normal neural histology with mild fibrosis around the nerve, which was associated with exploratory surgery. The fibres and axons were less organised and the number of fibres and axons was decreased in the middle (engrafted) and distal parts of the nerve in ANG group. The histologic features of the proximal nerve sections were similar to the sham treated animals. In SIC intestinal tube treated animals, the ensheathed fibrotic connective tissue involved neural elements in the middle sections. All layers of mucosa, submucosa and serosa of the small intestine segment and poorly regenerated nerve in the conduit were distinctive in histologic sections. Interestingly, fibres and axons were observed to be more organised after regenerative tissue traversed through the conduit in distal areas compared to middle areas (Figure 4, 5). The quantitative morphometric analysis of the nerve regeneration in the study groups is summarised in Table 1. Although the ANG group presented a significantly greater number of axons, increased axon diameter and myelin sheath thickness compared to SIC (p<0.05) in the middle and distal sites, quantitative nerve regeneration was also improved within the intestinal conduit.

### Immunohistological results

**Figure 6:**
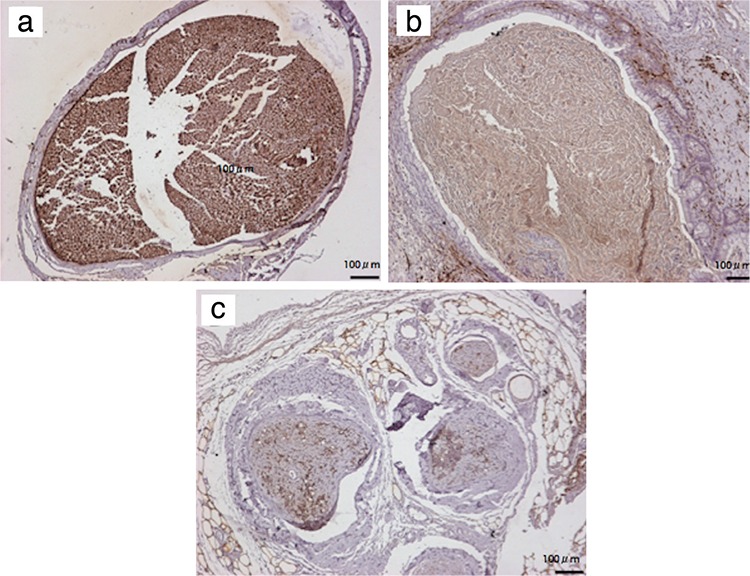
S-100 expression in the sections from proximal stump (a), intraconduit (b) and distal stump (c). Note that surrounding adipose tissue and neural elements in the subintestinal layer were also stained. Magnification at 10X, (Scale bar=100 μm).

**Figure 7:**
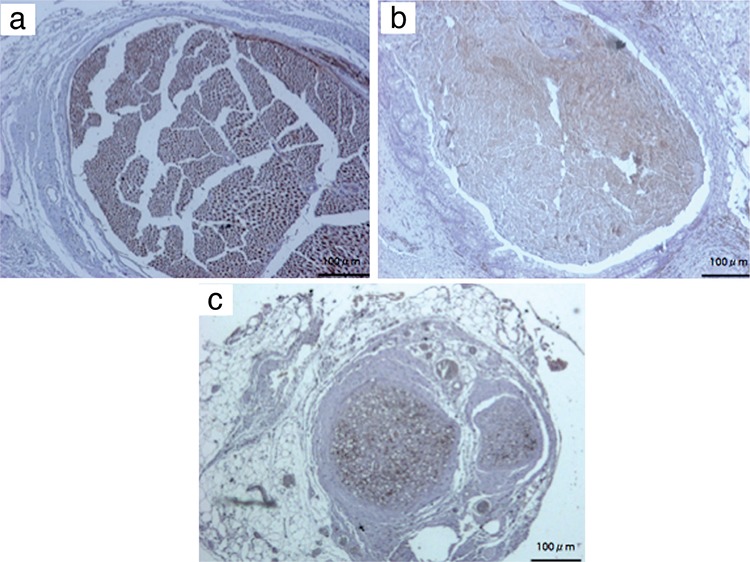
Neurofilament expression in the sections from proximal stump (a), intraconduit (b) and distal stump (c). Note that the tangled staining is arranged into a more regular fascicular staining pattern at the distal stump after traversing small intestinal conduit. Magnification at 10X (Scale bar=100 μm).

The results of immune staining for S-100 and neurofilament showed various outcomes. In the sham group, there were unique staining patterns observed in the proximal, middle and distal sections. However, the expression of S-100 and neurofilament was decreased in the middle and distal segments of the ANG and SIC groups. The staining was more evident in the SIC group (Figure 6, 7).

## DISCUSSION

Several conduit alternatives such as autologous or synthetic nerve grafts have been proposed for the repair of peripheral nerve gaps (3). Many synthetic biocompatible conduits, promising good results for neural regeneration, are commercially available (3,5). Foreign body reaction and lack of microenvironment has limited the clinical use of synthetic materials in the past. Better functional and bioabsorbable ones have been developed recently; however, these accomplishments did not discourage researchers from employing autologous tissues as nerve conduits (3,4,8,10) because autologous materials are easy to obtain, have no expense by means of operative charges, and synthetic biocompatible conduits are also suitable for only relatively short nerve defects (5).

The most popular approach is tubulisation of hollow vascular grafts to guide the axonal regeneration because of their large availability (1,17). Although thick-walled arteries are an ideal conduit for nerve defects, due to donor-site morbidity, sacrificing major arteries is unacceptable and their use is limited in clinical practice (1,3). Therefore, the focus of the investigations has shifted to the use of veins. Veins are more available than arteries and can be harvested with less donor-site morbidity. Veins also provide an appropriate microenvironment for axonal regeneration (3). However, the clinical application of veins is usually limited to bridging short nerve gaps and has not yet been fully accepted due to the tendency of thin walled veins to collapse and block regeneration (1,3,17). Thus, filling the lumen with supporting tissues (13,17) or cells may prevent collapse. Although the most preferred approach is filling the vein conduit with skeletal muscle (13,17,18), many other tissue fillers have also been addressed by several studies (19,20,21,22,23). However, not all supporting tissues have yielded proper regeneration and some may hinder axonal sprouting (24).

In addition to being a filler material for biological conduits, skeletal muscle itself has been employed as a solitary conduit for bridging nerve gaps (9). The muscle auto-grafts are abundant in individuals and display a longitudinally oriented basal lamina similar to peripheral nerves (13,17). However, fresh muscle is not suitable for immediate grafting because it contains muscle fibres that direct the nerve fibres to grow in different directions (7,8). Therefore, the muscle fibre must be processed by denaturation techniques to obtain empty scaffolds (10,11). The disadvantage of the denaturation process is shrinkage of the muscle tissue by up to 50% (10,11). Additionally, the muscle scaffolds are fragile after freezing, which makes their use difficult (10,11). To overcome the issues of muscle autografts, a rolling epimysium conduit that contains a few muscle fibres was recommended. However, this method was found to be less effective than ANG (4).

Another previous attempt to utilise native neural tissue is using epineurial sheaths. The epineurium itself provides less donor site morbidity and the appropriate microenvironment permitting nerve regeneration. However, epineurial conduits were found to be more suitable for short gaps than longer ones (4,12).

SIS containing a multilaminar middle layer of the small intestine was used as an alternative neural conduit for axonal regeneration (14,15,16,25). However, the tubularised full thickness small intestine itself was not used before.

SIS includes a variety of growth factors and ECM elements that support cell attachment and proliferation (26,27). Hadlock et al. (14) first reported the in vivo regenerative capacity of Schwann cell seeded SIS in their study. Su et al. (25) evaluated the biocompatibility of SIS with co- d Schwann cells in vitro and demonstrated that SIS supported the survival and proliferation of Schwann cells on its surface.

Similar supportive data were obtained from other studies of SIS extracts and demonstrated that extracts contain many types of nerve growth factors that stimulate neural growth on cultured pheochromocytoma cells (16,28). However, in a preliminary study, a 10 mm nerve gap was not completely regenerated through the SIS graft *in vivo*, and the sciatic nerve only grew into the proximal portion of the graft at 90^th^ days (15). Nerve regeneration becomes more evident in SIS if the defects between proximal and distal stumps are less than 10 mm (14,16). Data regarding the effectiveness of SIS are still lacking and there are no comparisons with other conduits (29).

SIS is a commercially available product that was FDA approved in 2003 (29). However, in experimental studies on neural regeneration, the investigators have preferred to prepare SIS grafts in their own facilities (14,15,25). Hadlock et al. (14) applied the rat SIS to bridge a sciatic nerve gap in rats, and stated that it was not firm enough to prevent luminal collapse and also inhibited further axonal regeneration.

Porcine SIS is thicker and has greater mechanical strength than rat SIS and may be more suitable as a scaffold in peripheral nerve tissue engineering (15). Unfortunately, using SIS in a xenograft could trigger a potential adverse immune response elicited by cell membrane epitopes and xenogeneic DNA (27). A multistep protocol is required to decellularise and reduce the antigenicity of SIS (26).

In this study, a tubularised SIC including all layers of the small intestine was harvested from the ileum and successfully used to bridge a 10 mm sciatic nerve defect without additional procedures. The results were supported with histologic data and also correlated with functional improvement based on SFI measurements. Proximal, middle and distal sections of the SIC were examined by immunostaining. The results allowed us to demonstrate the progress of neural regeneration. We found a stronger staining intensity of S-100 expression. Histological examination suggested myelinisation in the proximal nerve stump that decreased gradually in the middle and distal parts of the nerve. Based on neurofilament staining, the fibre distribution was irregular while traversing through the conduit; however, the axon fibres become reorganized in mini fascicles in the distal parts of the nerve. The axonal regeneration in SIC was inferior to the ANG and sham groups. This result can be explained by the limited regenerative capacity of the SIC and it was previously demonstrated that hollow tubes devoid of co-implanted cells induced only limited regeneration (1). Thus, better outcomes can be obtained if the conduit is filled with growth permissive substrates, neurostimulatory ECM, or neurotrophic agents, or is seeded with support cells, as shown in previous experimental studies (2,3,17).

Despite the encouraging results of SIC, it is not an alternative to ANG or other conduits in its current design. Furthermore, the surgical resection of a small intestine segment might be perceived as an invasive procedure for peripheral nerve reconstruction, when considering that donor sites such as the artery, vein, muscle etc. are generously available. However, in a multi-trauma patient that is necessitating abdominal exploration, SIC could be a good alternative at that point. We also believe that SIC can be employed for longer neural gaps compared to other biological and synthetic conduits. Moreover, its size can be adjusted according to the diameter and length of the injured nerve. With the aid of minimally invasive techniques, such as laparoscopy or natural orifice transluminal endoscopic surgery, small intestine could be harvested without a visible donor-site scar and morbidity.

Moreover, in humans, remnant intestinal tissue i.e. vermiform appendix could also be used instead of the small intestine. As rats do not have an appendix anatomically, the small intestine has been used in the experimental setting. However, due to ethical responsibilities, a limited number of animals were used and the experimental nature of the study is its limitations. Obtained data should not directly adapt to human being as well.

In conclusion, we hypothesise that the mucosal surface can act as basal lamina or can provide an appropriate microenvironment for neural tissue regeneration. Small intestines are abundant, easy to use and do not require physical or chemical processing without donor-site morbidity. The present study showed that neural regeneration could be accomplished through autologous tabularised intestinal segment. The vermiform appendix could be a good alternative. However, further basic research is required before these conduits can be used in clinical practice.

## Figures and Tables

**Table 1:**
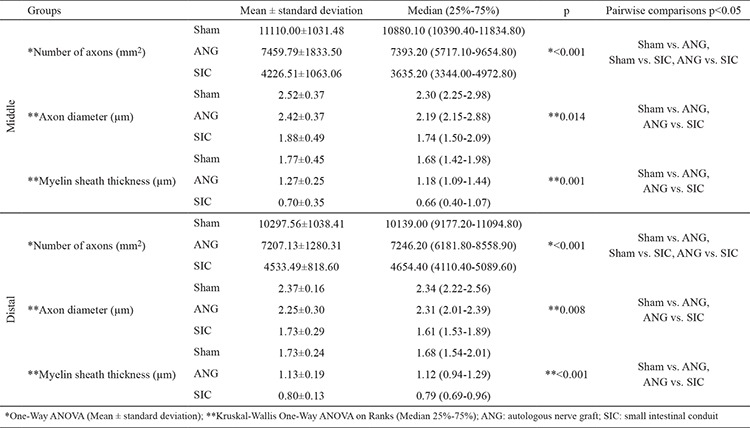
Histomorphometric analysis results at 12 weeks
